# Supplementary material for: A Novel Method to Reduce ELISA Serial Dilution Assay Workload Applied to SARS-CoV-2 and Seasonal HCoVs
Source: Viruses. 2022 Mar 9;14(3):562. doi: 10.3390/v14030562 (PMC8955134; doi:10.3390/v14030562)
Supplement: Supplementary file 1 [file viruses-14-00562-s001.zip › viruses-1582554 supplementary.docx]

## Supplementary Material


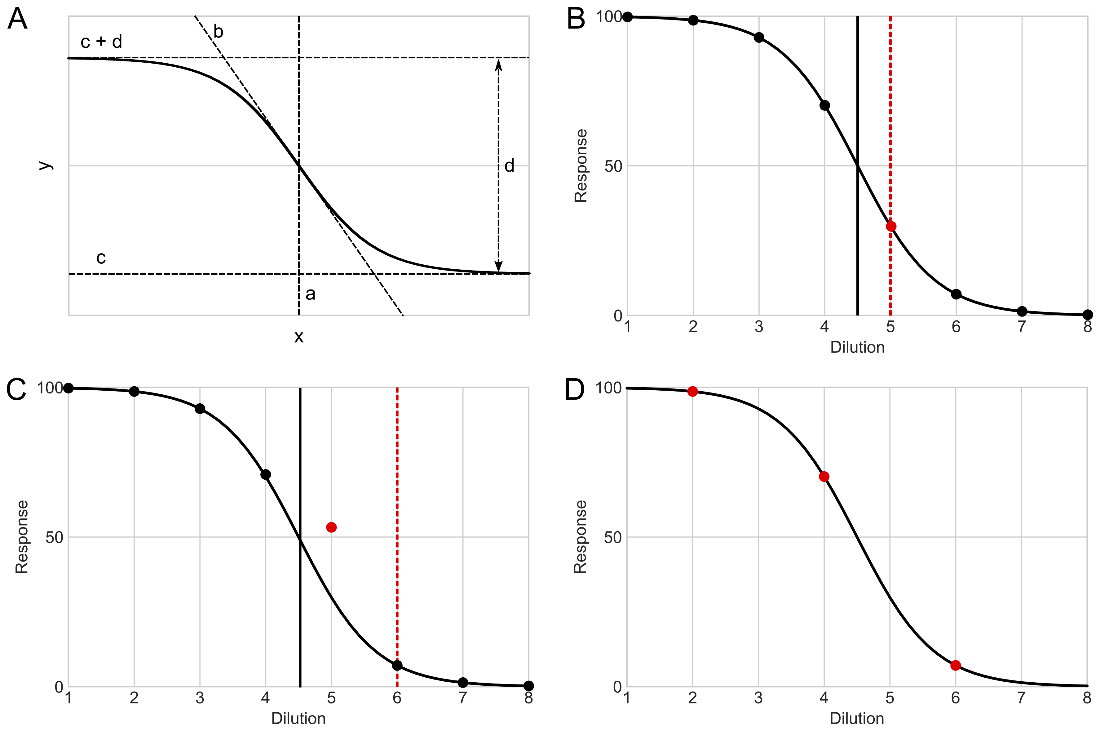


**Fig. S1 (A) Four parameter sigmoid function.** y = c+d/(1+exp-b(x-a)). a defines the horizontal location of the curve on the x axis. b sets the gradient of the curve. c sets the minimum y value, and d sets the distance between the minimum and maximum y value. The x-axis shows the index of the dilution series. **(B) Discretizing reduces precision.** The first dilution underneath a 50% response (red point) is 5, but the true inflection point is 4.5 (black vertical line). **(C) Overreliance on individual measurements.** The measurement at dilution 5 is erroneous, so the first dilution underneath a 50% response is 6 (red dotted line), but the true EC50 is 4.5 (black line). **(D) Sigmoid curves can be fit with fewer dilutions than are commonly conducted.** Statistics of interest (e.g. inflection points or endpoints) can then be computed from the curve. Hierarchical modelling of multiple samples that share curve characteristics improves the inference of individual samples.

###

###
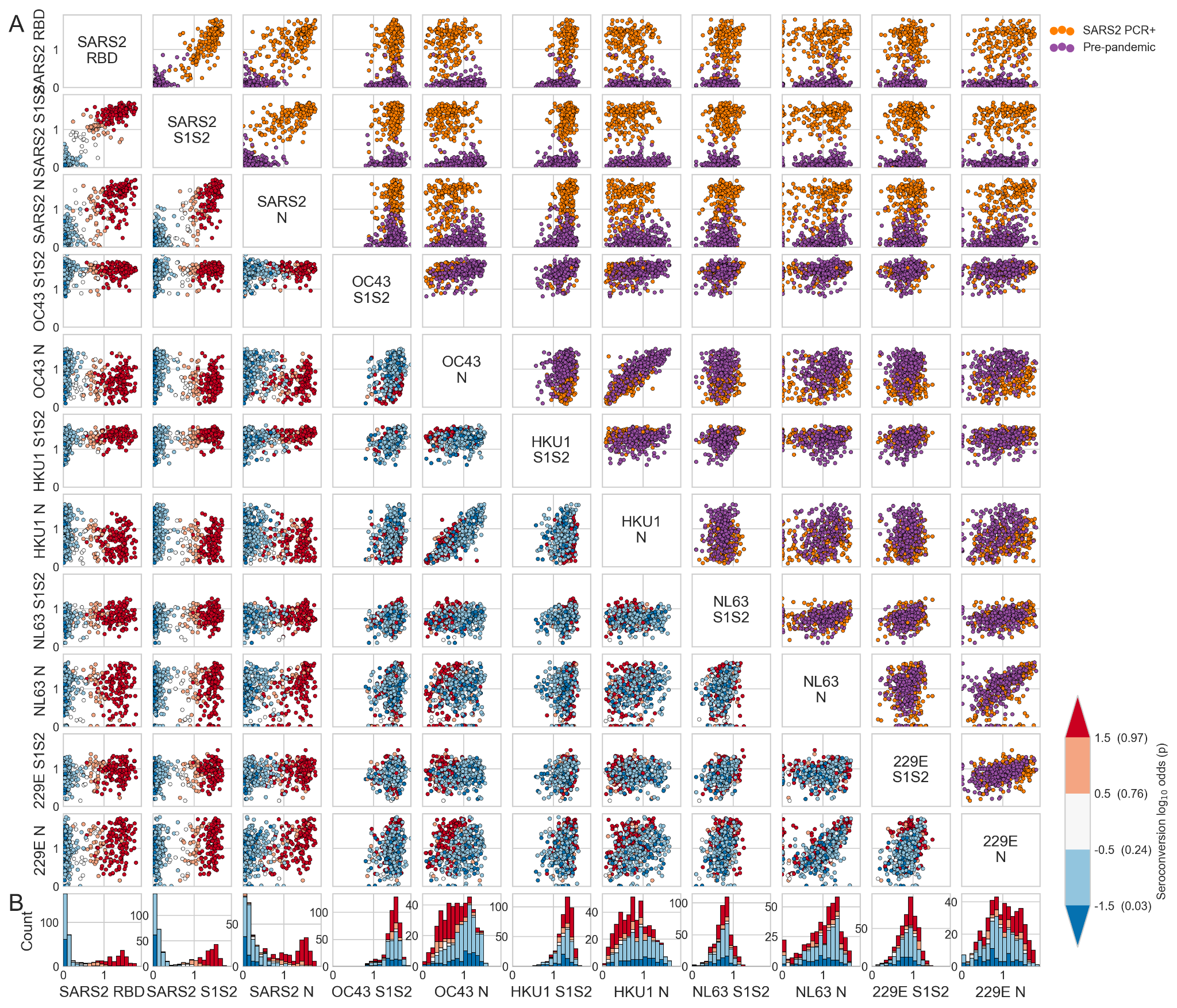


**Fig. S2. Reactivity to SARS-CoV-2, OC43, HKU1, NL63, 229E spike and N antigens in pre-pandemic and SARS-CoV-2 PCR-positive human serum samples. (A)** Scatter-matrix of ELISA OD measurements for 1:40 diluted serum samples. Above the main diagonal colors indicate whether a sample was collected before the pandemic or from a SARS-CoV-2 PCR-positive individual. Below the main diagonal, colors indicate SARS-CoV-2 seroconversion log odds. **(B)** Histograms display univariate distributions in each column and are colored by seroconversion log odds.


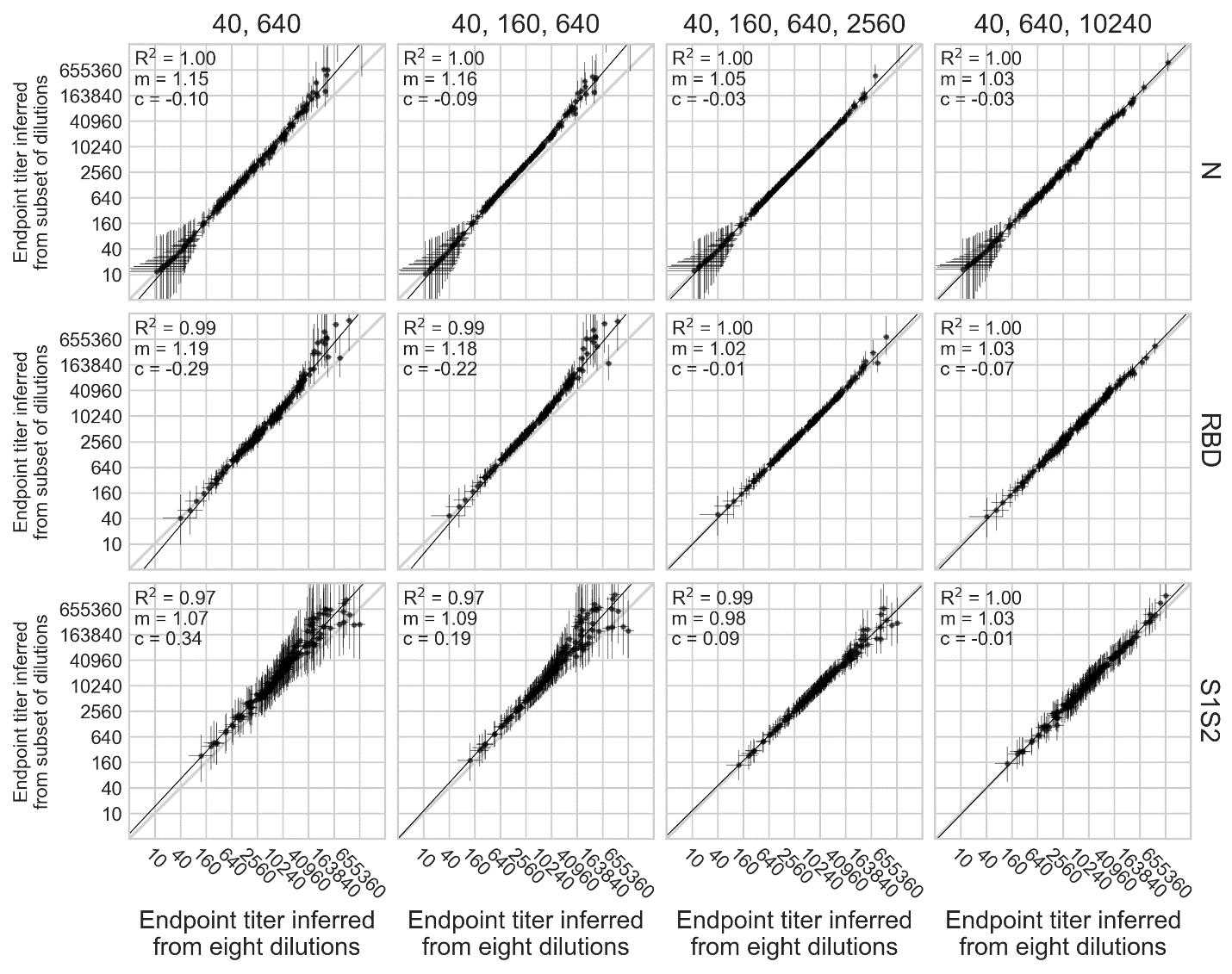


**Fig. S3. Inferring endpoint titers from minimal dilution series.** As Fig. 3, except here we performed the analysis for an arbitrarily chosen endpoint titer, defined here as the serum concentration that would yield an OD reading of 0.1.

###
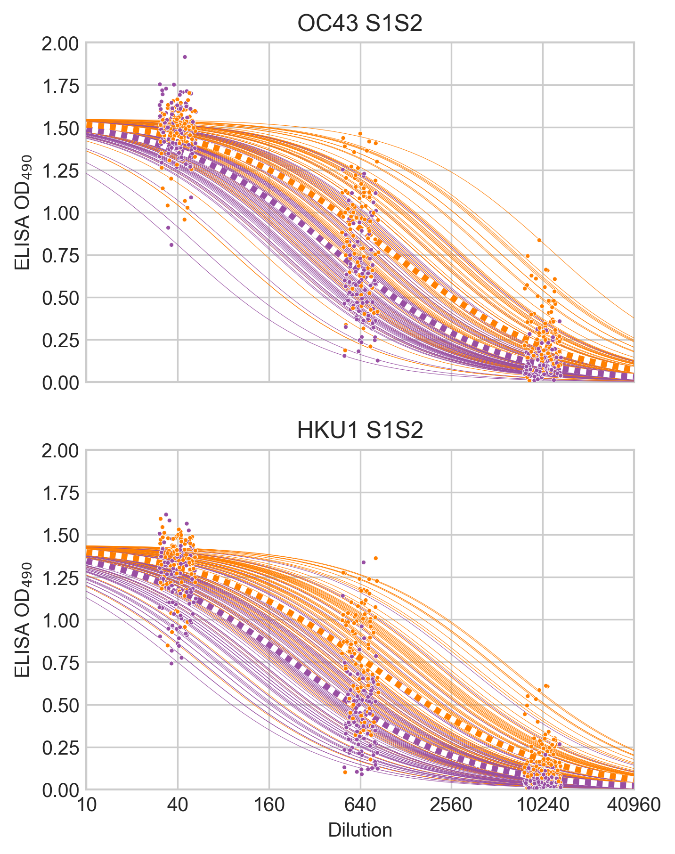


### Fig. S4. Dilution series of OC43 and HKU1 S1S2. Thin solid lines show sigmoid curves for pre-pandemic (purple) and PCR-positive (orange) samples fit to OD values from their dilution series (points). Thick dashed lines indicate the mean curve of each group. A small amount of x-axis jitter has been added to the points to prevent overplotting. Curves are drawn using the mean of the parameter posterior distributions. The difference between groups for OC43 S1S2 when only looking at OD values from the 1:40 dilution (Fig. 2) is substantially smaller than the difference between the inflection titers (Fig. 5). This is caused by the OD measurement becoming saturated at 1:40. Similarly, for HKU1 the difference between groups is smaller at the single dilution of 1:40 (Fig. 2) than it is when comparing inflection titers (Fig. 5).

### Table S1. Summary of human serum panels.

| Panel | Description | n |
| --- | --- | --- |
| Pre-pandemic | Obtained before emergence of SARS-CoV-2, between 2013 and April 2019 by the Institute for Influenza Research, UW Madison. | 248 |
| UWH PCR positive | PCR-positive for SARS-CoV-2 from University of Wisconsin Hospital. | 188 |
| Commercial positive | SARS-CoV-2 positive antisera purchased from Lampire Biological Laboratories. | 12 |
| EGH early infection | Paired samples collected from SARS-CoV-2 patients soon after diagnosis at the Eiju General Hospital. | 44 |
| EGH late infection | Paired samples collected from SARS-CoV-2 patients late during infection at the Eiju General Hospital. | 44 |
| MCRI | Samples collected after the emergence of SARS-CoV-2 from participants of a community cohort study by the Marshfield Clinic Research Institute. | 162 |
|  |  |  |
